# My Reasons for Living: A Descriptive Study of the Motives for Not Committing Suicide Among Patients Diagnosed With Schizophrenia

**DOI:** 10.7759/cureus.64092

**Published:** 2024-07-08

**Authors:** Yvonne Flores Medina, Ricardo Saracco-Alvarez, Mauricio Rosel Vales, Luis G Moncayo-Samperio, Cesar Celada Borja, Alejandra Mondragón Maya, Ana Seubert Ravelo, Jesús Luna Padilla, Erik Morelos Santana, Lenin Pavón

**Affiliations:** 1 Investigaciones Clinicas, Instituto Nacional de Psiquiatría Ramón de la Fuente Muñiz, Mexico City, MEX; 2 Servicios Clínicos, Instituto Nacional de Psiquiatría Ramón de la Fuente Muñiz, Mexico City, MEX; 3 Departamento de Psicogeriatría, Instituto Nacional de Psiquiatría Ramón de la Fuente Muñiz, Mexico City, MEX; 4 Servicios Clinicos, Instituto Nacional de Psiquiatría Ramón de la Fuente Muñiz, Mexico City, MEX; 5 Facultad de Estudios Superiores Iztacala, Universidad Nacional Autónoma de México, Mexico City, MEX; 6 Laboratorio de Neuromodulaición, Instituto Nacional de Psiquiatría Ramón de la Fuente Muñiz, Mexico City, MEX; 7 Subdirección de Investigaciones Clinicas, Instituto Nacional de Psiquiatría Ramón de la Fuente Muñiz, Mexico City, MEX

**Keywords:** reasons for living, schizophrenia, suicidal risk, resilience, protective factor

## Abstract

Background and objective

Reasons for Living (RFL) constitute a construct that enables identifying the reasons for not committing suicide. These reasons are based on significant aspects of life, on the commitment to some ideals which may inhibit the impulse of committing suicide. The present study aimed to explore the RFL in a sample of patients with chronic schizophrenia; analyze the association of RFL with the duration of illness, previous suicide attempts, hospitalizations, and schooling; and describe the potential differences between male and female patients in this context.

Materials and methods

A total of 94 patients with schizophrenia were assessed. The Reasons for Living Inventory (RFLI) was applied and a structured interview for clinical and sociodemographic data was performed to gather data. Frequencies and descriptive statistics were calculated, and Spearman's correlation analysis was employed.

Results

The mean score among the sample was 3.9, with 3.8 as the cut-off point under which the presence of suicide risk is significant. The RFLs indicated as most important by patients were those in the domains of Survival and Coping Beliefs and Responsibility to Family. Non-significant differences were observed between groups. An association was observed in terms of age, duration of illness, number of hospitalizations, and RFLI scores.

Conclusions

The sample in the present study obtained high scores in the RFL domain of Survival and Coping Beliefs and low scores in the domain of Fear of Suicide, reflecting a specific response pattern that contrasts with other high suicidal-risk populations. We suggest that this construct could represent a protective factor for schizophrenia patients, including chronic patients with previous suicide attempts and high hospitalization rates, which were common variables observed in our clinical sample.

## Introduction

According to global estimates from the World Health Organization, more than 700,000 individuals commit suicide each year, making suicide the fourth major cause of death in young adults [1]. It has been reported that being diagnosed with any mental condition, including schizophrenia, constitutes a risk factor for suicidal behavior, regardless of age, gender, or geographical location [2]. Schizophrenia is a psychiatric disorder associated with progressive physical and mental decline. Moreover, it significantly impacts the health and finances of the patients’ families [3]. It has been reported that 20-40% of schizophrenia patients have attempted suicide in their lifetime, especially in the first year after diagnosis, and up to 10% of diagnosed schizophrenia patients have committed suicide [4]. The suicidality risk in schizophrenia may be guided by several factors with different risk rates, such as the age of onset, chronicity [5], poor adherence to treatment, higher education, substance use, previous suicide attempts, hospitalizations, and depression [6].

It is noteworthy that a considerable body of research regarding suicidal behavior in schizophrenia has focused on the description of its risk factors and prevention strategies [7-11], while significantly less attention has been paid to elucidating the potential protective factors against suicide [12,13]. The protective factors in this sense are defined as personal and psychosocial conditions that diminish an individual’s probability of displaying suicidal behaviors [2]. Such factors may be related to treatment, support networks, or individual biological and psychological characteristics [14]. In schizophrenia patients, elements like social support, coping abilities, life satisfaction, and personal recovery have been identified as protective factors [12,13], but the magnitude of protection they provide is not conclusive.

Reasons for Living (RFL) constitute another set of protective factors that have been identified in high-suicidality populations [15,16]. This construct enables identifying the reasons for not committing suicide. The reasons are based on significant aspects of life, on the commitment to some ideals that may inhibit the impulse of committing suicide or resistance attitudes towards this behavior due to fear [2,14]. RFL has been inversely correlated with depression symptoms (-0.27, p=0.05), hopelessness (0.23, p=0.5), and suicidal ideation (-0.44, p=0.5) in other psychiatric populations including patients with depressive disorder [14]. RFL has been proposed as a variable that may “make the difference” between suicide ideation and attempt [15].

At least, two empirical research lines have supported RFL as a significant protective factor: (1) RFL is inversely correlated to suicidal ideation in general and clinical populations among teenagers and adults; and (2) RFL is associated with lower levels of depression (-0.33, p=0.001), entrapment (-0.27, p=0.001), and low self-esteem (-0.16, p=0.001 [16]. The construct has been mostly explored with an instrument called the Reasons for Living Inventory (RFLI), designed by Marsha Linehan [17]. It includes the following domains: Survival and Coping Beliefs, Responsibility to Family, Children-related Concerns, Fear of Suicide, Fear of Social Disapproval, and Moral Objections. RFL has shown acceptable internal consistency (Cronbachs α=0.72-0.92), as well as test-retest reliability (0.75-0.85).

The study of RFL in schizophrenia has two clear precedents. The first one is the paper titled A Study of Quality of Life and Reasons for Living in Patients Suffering From Chronic Mental Illnesses [18], in which the authors describe the most important RFLs in male patients diagnosed with schizophrenia, bipolar disorder, depression, or alcohol use disorder, i.e., Survival and Coping Beliefs and Responsibility to Family; and females, i.e., Fear of Suicide. The second constitutes a study by Hocaoglu and Babuc [19] that stated that patients with schizophrenia who report suicidal ideation identify fewer RFLs, and pointed to the negative correlation between RFLI scores and the Calgary Depression Scale for Schizophrenia (CDSS) as well as Positive and Negative Syndrome Scale (PANSS)-Negative scores.

There is scarce literature exploring RFLs in patients with schizophrenia. The exploration of a set of beliefs that buffer individuals from suicidality in the face of stressors even in circumstances of loss, social distress, or other life challenges, could help us to delve deeper into the aspect of resilience as a bulwark against suicidal ideation [14]. In light of this, the present study aimed to explore RFLs in a sample of patients with chronic schizophrenia; analyze the associations with duration of illness, previous suicide attempts, hospitalizations, and schooling; and describe potential differences between male and female patients in this context.

This article was previously posted to the Research Square preprint platform on September 27, 2022: https://doi.org/10.21203/rs.3.rs-2093806/v1; PPR: PPR551127.

## Materials and methods

Study design and setting

We employed a cross-sectional descriptive design to conduct this study. The assessment was approved by the Research Ethics Committee of the Instituto Nacional de Psiquiatría Ramón de la Fuente Muñiz (INPRFM) with registry number CEI/C/004/2019, as part of a broader research project named “Non-pharmacological treatments for schizophrenia patients”; the study was conducted between 2022 and 2024.

Participants

Convenience sampling was used to recruit participants. Ninety-four patients diagnosed with schizophrenia according to the Diagnostic and Statistical Manual of Mental Disorders, Fifth Edition (DSM-5 criteria), were recruited at the INPRFM, in Mexico City. All participants were at least 18 years old, had a minimum of six years of formal education, were undergoing pharmacological treatment, and were clinically stable (no hospitalizations or medication modifications in the last three months) at the time of the assessment. Exclusion criteria included any comorbid neurological condition, substance use disorder (except for nicotine), clinical diagnosis of intellectual disability, or presence of any sensorial or motor disability that could interfere with the assessment. 

Tools

The following demographic and clinical data were obtained using a structured interview: age, years of education, age of onset, illness duration, hospitalizations, suicide attempts, and pharmacological treatment.

RFLI

RFLI is a self-report instrument consisting of 48 items with ratings on a Likert scale ranging from 1 (not at all important) to 6 (extremely important). Each item explores the relevance of distinct reasons for not committing suicide as scored by the patient. The cut-off point (gold standard) of the instrument’s original version is 3.8/6 points, meaning that scores under 3.8 are indicative of the presence of suicide risk [17]. The RFLI includes six domains: Survival and Coping Beliefs (e.g., can find other solutions to problems); Responsibility to Family (e.g., my family depends on me); Children-related Concerns (e.g., I want to watch my children as they grow); Fear of Suicide (I am afraid that my method of killing myself would fail ); Fear of Social Disapproval (e.g., other people would think I am weak and selfish); and Moral Objections (e.g., my religious beliefs forbid it). Cut-off points and means per domain analyses have been reported in Latin-American versions [20,21]. The RFLI Mexican validation can be found in the study by Villela [22]; for this version, the alfa de Cronbach was 0.02, and the factor analysis using six factors showed an index of 0.60, which is considered moderate.

Procedure

Candidates were invited to enroll in the study. The clinical diagnosis of schizophrenia was made by a specialized psychiatrist as per DSM-5, and the assessment of the severity of symptoms was using PANSS. After verifying the inclusion/exclusion criteria, participants signed an informed consent letter that was approved by the institution’s Research Ethics Committee. Then, research assistants in the psychology master’s degree program and the lead investigator obtained the sociodemographic information and administered the scale.

Statistical analyses 

Frequencies and descriptive statistics were calculated, including the mean and standard deviation (SD) for each domain and the RFLI total score. The Mann-Whitney U test was used to explore the differences between the scores of women and men, and Spearman correlation analysis was used to explore the association between RFLI’s six domains and years of education, chronicity (years), number of hospitalizations, and suicide attempts. JASP 0.18.1 was used for all statistical analyses.

## Results

A total of 94 participants were included; of them, 28.7% (n=27) were women and 71.3% (n=67) were men. The demographic and clinical information of the cohort are presented in Tables 1-2.

**Table 1:** Sociodemographic characteristics and medical history of the sample (n=94) PANSS: Positive and Negative Syndrome Scale

Variables	Mean	Std. deviation	Minimum	Maximum
Age, years	36.8	10.4	18	61
Years of education	12.9	2.7	6	18
Age of onset, years	25.1	7.5	15	47
Duration of illness, years	12.1	7.9	.5	38
Number of hospitalizations	0.55	.99	0	6
Suicide attempts	0.33	.75	0	4
PANNS total score	71	12.5	44	93

**Table 2:** Pharmacological treatment in the cohort

Medication	N	%
Amisulpride	1	1.1
Fluoxetine	1	1.1
Haloperidol	4	4.3
Paliperidone	4	4.3
Sulpiride	5	5.3
Clozapine	13	13.8
Olanzapine	14	14.9
Aripiprazole	25	26.6
Risperidone	27	28.7

**Figure 1:**
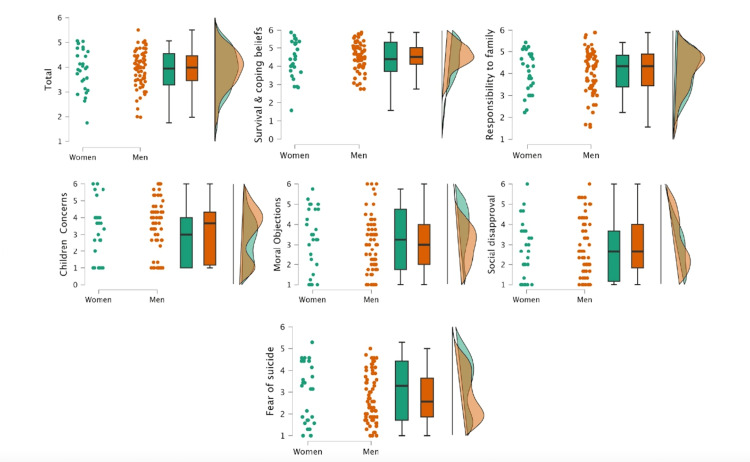
Differences between women and men in the various domains of the Reasons for Living scale No significant differences were found between women and men. Reason for Living total mean: W=875, p=0.08; Survival and Coping Beliefs: W=863, p=0.73; Responsibility to Family: W=870, p=0.77; Children-related Concerns: W=811, p=0.43; Moral Objections: W=967, p=0.60; Fear of Social Disapproval: W=790, p=0.34; and Fear of Suicide: W=1011, p=0.37

**Figure 2:**
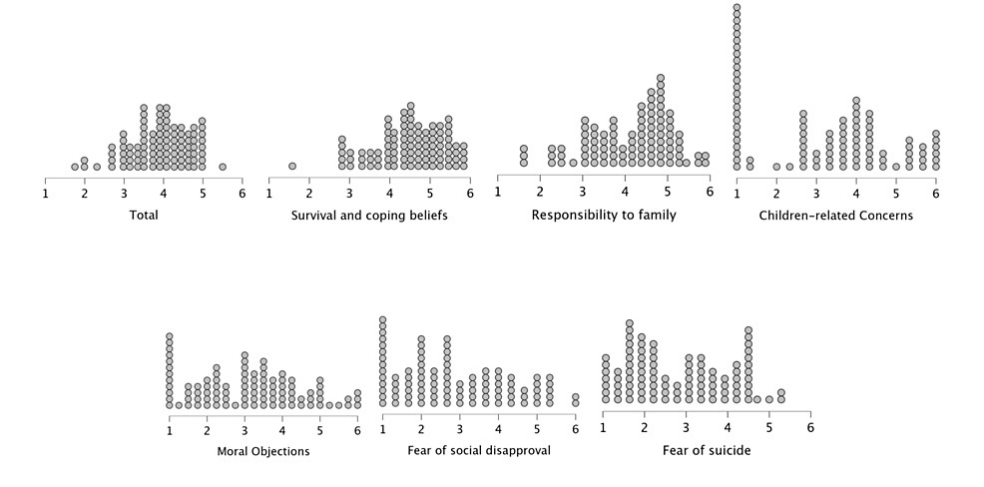
Response Frequency in each of the Reasons for Living domains Measured on the Likert punctuation scale from 1 (not at all important) to 6 points (extremely important). The total mean: 3.9 (SD: 0.7); Survival and Coping Beliefs: 4.4 (SD: 0.8); Responsibility to Family: 4.1 (SD: 0.9); Children-related Concerns: 3.2 (SD: 1.6); Moral Objections: 3.1 (SD: 1.3); Fear of Social Disapproval: 2.8 (SD: 1.4), and Fear of Suicide: 2.8 (SD: 1.2)

Figure 1 illustrates the frequency of responses, with scores ranging from 1 (not at all important) to 6 points (extremely important): the mean and SD of the total and each of the RFLI subscales. Considering the cut-off point of 3.8, under which the presence of suicide risk is significant, the sample mean score can be regarded as within borderline parameters (3.9). The RFLs indicated as most important by patients were those in the domains of Survival and Coping Beliefs and Responsibility to Family. Fear of Suicide and Fear of Social Disapproval were the least relevant RFLs in the studied sample. Non-parametric Mann-Whitney U test was performed to compare the scores of men and women in the RFL domains; non-significant differences were observed between groups (Figures 1, 2).

Statistically significant correlations were observed between years of education, hospitalizations, and suicide attempts with several subscales of RFLI; the Spearman coefficients are shown in Table 3.

**Table 3:** Mean Reasons for Living Inventory scores in schizophrenia patients and Spearman's correlation with the clinical data Spearman's Rho, age-adjusted *p<0.05, ** p<0.01, ***p<0.001

Reasons for Living Inventory	Years of education	Duration of illness	Number of hospitalizations	Suicide attempts
Total	-.091	0.139	-0.274	-0.222*
Survival and coping beliefs	-.009	0.133	-0.286	-0.305*
Responsibility to family	-0.007	0.166	-0.194	-0.153
Children-related concerns	-.288*	-0.018	-0.145	-0.118
Moral objections	-0.08	0.171	-0.268*	-0.098
Fear of social disapproval	0.029	0.003	0.036	-0.056
Fear of suicide	-0.13	0.139	-0.031	0.072

## Discussion

The present study aimed to describe the RFL in a sample of chronic schizophrenia patients. The results indicate that although the total mean score of RFLI in the sample reached borderline suicide risk scores, clinically stable patients were able to identify a significant number of reasons for not committing suicide. In our sample, the reasons most frequently identified as important corresponded to the Survival and Coping Beliefs domain, which represents the patient’s confidence in conducting themselves effectively through difficult circumstances. This domain includes items such as “I believe I can learn to adjust or cope with my problems”; “I believe I have control over my life and destiny”; and “I have the desire to live”. These sets of beliefs can also be found in the literature on resiliency in an overarching category referred to as cognitive ability, which includes attributional styles, coping and problem-solving, cognitive process biases, and emotional management [23].

The second most important reason recognized in our sample is the Responsibility to Family, which pertains to the following assumptions: “My family might believe I did not love them”; “I have a responsibility and commitment to my family”. This domain additionally showed a significant correlation with age and time of onset of the disease. In another clinical population, i.e., patients with severe depression, it has been observed that a higher score on this construct correlates positively with hopelessness and increases the severity of suicidal ideation, or inversely correlates with suicide attempts [24]. These differences are particularly important because they suggest that different patterns of RFLs can be observed across conditions of severe mental disorders. In the case of patients with schizophrenia, the family represents a primary support system, and the relationship with them is associated with the magnitude of suicidal risk in these patients [25].

The least important reasons corresponded to the Fear of Suicide and Fear of Social Disapproval domains. These results are consistent with the report of Yella et al. [18] among a clinical sample of patients with different psychiatric conditions including schizophrenia, bipolar disorder, depression, and alcoholism. Again, we can observe differences in the response pattern compared to other clinical populations. For instance, the high scores in Survival and Coping Beliefs, and low scores in Fear of Suicide reflect a contrasting response pattern compared with borderline personality [17]. Unlike the findings of Yella et al. [18], we did not observe any statistical differences between males and females in the RFLI scores. We believe that this difference may be attributed to our sample only comprising patients with schizophrenia, in contrast to the studies that include several psychiatric conditions. Another important factor is that our sample predominantly consisted of men.

The buffering hypothesis describes the protective factors as an internal psychological construct that attenuates the risk of the fatal outcome in high levels of suicidality. The inverse association observed in our study between RFL total and Survival and Coping Beliefs domain with suicidal attempts is consistent with that reported in several samples including clinical and healthy samples, adolescents, adults, and the elderly [20,21]; it suggests that this construct could represent a resilience factor for schizophrenia patients, including chronic patients with previous suicide attempts and high hospitalization rates. The negative correlation of Moral Objections with the number of hospitalizations aligns with the studies suggesting that this factor is associated with a lower number of suicide attempts [25,26]. Targeted interventions that focus on reinforcing RFLs, specifically on those that patients indicate as most relevant, such as family or problem-solving skills, can enable individuals to consider their motivations and make a difference in the internal debate about the reasons to live vs. the reasons to die [23,27].

Our study has certain limitations. Primarily the descriptive nature of the research did not allow us to formulate a definitive conclusion about the protective nature of RFL against suicidal behavior in schizophrenia. We propose that this can be a potential new field of study and hope to lay the foundation for more ambitious research on this topic, given the current dearth of literature on this protective factor in this clinical population. Along these lines, distinguishing between groups of patients with high and low suicidality could offer a broader picture of this construct as a protective factor: Some studies indicate that RFLs tend to be a protective factor in individuals who have not made any suicide attempts, while other factors such as religious beliefs are more relevant in those who have made previous attempts [28].

Also, conducting studies across two critical stages of the disorder - the first year after receiving the diagnosis and maybe in advanced stages in the course - could offer deeper insights into the possible changes in the reasons that these people have for not committing suicide. Although 94 patients were evaluated, their clinical and sociodemographic characteristics were heterogeneous, which limits the generalization of these findings to the broader population. Hence, studies with larger samples could help us to identify other variables that may be related to the RFL construct. Finally, an additional limitation is that the socioeconomic status of the patients was not considered in our analysis.

## Conclusions

Men and women diagnosed with schizophrenia identify several reasons for not committing suicide. Among them, the most important, as documented based on RFLI, are Survival and Coping Beliefs and Responsibility to Family, while Moral Objections or Fear of Suicide are less important. This set of RFLs differs from other clinical populations with high suicidality and may represent a mediating factor between suicidal ideation and suicidal behavior in this population. Exploring new protective factors such as RFLs in the suicidal behavior of patients with schizophrenia may enable us to broaden the scope and opportunities for prevention given that despite enormous efforts, both in terms of pharmacological and psychosocial treatment, the number of suicides among these patients remains considerably high.

## Appendices

A factor analysis with varimax rotation was performed with the 48 items for RFLI (Table 4).

Kaiser-Meyer test: 0.677; Barlett's test: x^2^=2764.54, p=0.001; and X^2^=1163.55, p=0.001.

**Table 4:** Factor analysis with varimax rotation

	Factor 1	Factor 2	Factor 3	Factor 4	Factor 5	Factor 6
Item 20	0.738					
Item 19	0.665					
Item 17	0.642					
Item 44	0.619					
Item 22	0.614					
Item 24	0.614		0.531			
Item 42	0.591					
Item 40	0.590					
Item 1	0.569					
Item 12	0.555					
Item 4	0.542					
Item 13	0.531					
Item 16	0.530					
Item 35	0.514		0.413			
Item 5	0.505			0.450		
Item 10	0.491					
Item 37	0.463		0.507			
Item 9	0.455					
Item 25	0.405					
Item 47		0.676				
Item 38		0.620				
Item 27		0.616				
Item 48		0.606				
Item 26		0.584				
Item 46		0.582				
Item 43		0.580				
Item 41		0.575				
Item 34		0.572				
Item 30		0.563				
Item 28		0.545			0.439	
Item 39		0.442				
Item 36			0.609			
Item 18				0.657		
Item 23				0.618		
Item 33				0.442		
Item 7				0.405		
Item 21					0.763	
Item 11					0.751	
Item 8						0.626
Item 15						0.527
Item 29						0.489
Item 14						0.428
Item 2						
Item 3						
Item 6						
Item 31						
Item 32						
Item 45						
Proportion on variance	11.8	11.7	7.6	5.6	5.5	7.4
Eigenvalue	11.34	5.65	2.47	2.16	2.06	1.84
